# A Survey on Artificial Intelligence Aided Internet-of-Things Technologies in Emerging Smart Libraries

**DOI:** 10.3390/s22082991

**Published:** 2022-04-13

**Authors:** Siguo Bi, Cong Wang, Jilong Zhang, Wutao Huang, Bochun Wu, Yi Gong, Wei Ni

**Affiliations:** 1 Library, Fudan University, Shanghai 200433, China; fdbsg@fudan.edu.cn (S.B.); jlzhfd@fudan.edu.cn (J.Z.); 2 School of Life Sciences, Fudan University, Shanghai 200433, China; congwang@fudan.edu.cn; 3 Informatization Office, Fudan University, Shanghai 200433, China; 21307090024@m.fudan.edu.cn; 4 Department of Materials Science, Fudan University, Shanghai 200433, China; gongyi@fudan.edu.cn; 5 Commonwealth Scientific and Industrial Research Organisation, Sydney, NSW 2122, Australia

**Keywords:** Internet-of-Things, artificial intelligence, smart libraries

## Abstract

With the boom in artificial intelligence (AI) and Internet-of-Things (IoT), thousands of smart devices are interconnected with each other and deeply applied into human society. This prosperity has significantly improved public service and management, which were traditionally based on manual work. As a notable scenario, librarianship has embraced an era of “Smart Libraries” enabled by AI and IoT. Unlike existing surveys, our work comprehensively overviews the AI- and IoT-based technologies in three fundamental aspects: smart service, smart sustainability, and smart security. We then further highlight the trend towards future smart libraries.

## 1. Introduction

With the rapid development of artificial intelligence (AI) and Internet-of-Things (IoT), thousands of smart devices can be interconnected with each other [1,2,3,4,5,6]. A series of innovative concepts have emerged and penetrated into all aspects of human life, e.g., “Smarter Planet” [7], “Smart City” [8], “Smart Community” [9], and “Smart Campus” [10]. As a key and indispensable field, librarianship has become a convenient scenario aided by AI and IoT. Distinctive advanced AI-based approaches applied in libraries include, but are not limited to, natural language processing (NLP) [11], deep learning (DL) [12], recommender systems [13], machine vision [14], and smart acquisition [15]. Distinctive advanced IoT-based approaches applied in libraries include but are not limited to radio frequency identification (RFID) [16], near-field communication (NFC) [17], wireless fidelity (Wi-Fi) [18], bluetooth low energy (BLE) [19], and robotic systems [20]. As discussed in [21,22,23], there is still room for improvement in traditional librarianship on the efficiency of service when facing the diverse and growing needs for timely resource retrieval. Evidently, the inefficient issue of heavy reliance on human intervention can be alleviated. For example, AI-aided robots can keep costs low in consulting service. Specifically, in the trivial service for finding specific books, a smart robot with an NLP engine can efficiently understand the personalized needs of readers and generate optimal navigation for finding books, under real-time localization service with RIFD, Wi-Fi, and BLE beacons. This can remarkably improve the circulation efficiency for both librarianship and readers, especially in the case when the wanted books are randomly placed by the other readers, in contrast to the traditional manual circulation.

### 1.1. Traditional vs. Smart Libraries

**Figure 1:**
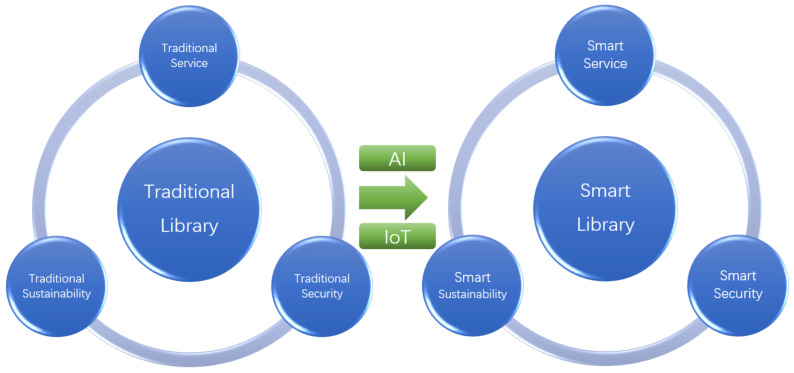
Transition from traditional to smart libraries via applying AI and IoT.

Traditional libraries are generally required for various services and managements. In a multi-step routine process of borrowing books, a reader has to enter a specific library for preserving books of interest, carry books to a specific place (i.e., circulation desk), show the librarian his/her identification for verification, and finally confirm the books to borrow. However, it is impractical to ignore the frequently occurred cases that the intended books have been borrowed by others during the previous procedure. A priori unaware of which branch stores the intended books, readers have to patronize several locations one by one. However, in the era of the “Smart Library”, obtaining the books only involves concise steps via a smart terminal device: confirm and make an appointment for the intended books, fetch the books under the smart guide and devices furnished by the library, e.g., the optimal schedule for the borrowing process. Circulation efficiency improves upon traditional methods, relying on the tremendous power of AI and IoT adopted by librarianship. Specifically, once the appointment and corresponding necessary information by readers are received by an AI engine, the smart schedule for the wanted books is initiated in a timely manner to smartly compute the optimal time and optimal branch library for delivery. It is necessary to make a reasonable decision on which branch library is the optimal one for delivery. Consider the frequent and general cases that on one hand, a due date for the wanted copies preserved in a nearby branch library is upcoming for a punctual borrower, judged based on the historical circulation data by the AI engine. On the other hand, the location of the branch library for preserving sufficient copies is considerably far from the appointment reader. The AI engine can thus recommend a reasonable schedule according to personalized needs for readers. In the stage for fetching the books, under the reasonable schedule by the AI engine, the reader can conveniently find the wanted books under the navigation provided by a Wi-Fi-based local positioning system combined with an RFID localization system [18], and fulfill the borrowing via an RFID-based self-borrowing-and-returning machine. In general, as discussed in [22], an objective of the smart library is always user-centric, meanwhile it can smartly capture the needs of users, and provide efficient resources and services to readers. As described in the above case, AI technology can smartly recommend a schedule for book delivery, and IoT technology can be utilized to smartly provide optimal navigation for the specific localization of wanted books [21,22,23,24,25]. The whole process can save time and space. Almost without any direct human intervention, this can be a suitable process, especially in the pandemic era of COVID-19 [26]. Specifically, the discussed application scenario is just a miniature of the smart service aspect reflected in the “Smart Library”. Compared to the traditional library [27], people can mainly benefit from the library development as driven by AI and IoT in three categories: smart service, smart sustainability, and smart security; see Figure 1.

### 1.2. Motivation

It is noteworthy that the library is not merely a place of leisure for reading and studying, but as a foundation for accelerating the progress of civilization development. In addition, it is precisely the vigorous promotion of AI-aided IoT to make the library smarter and more efficient. Thus, it urgently needs a comprehensive survey for such a key and extremely promising domain, namely, the AI-aided IoT applied in a “Smart Library”.

Existing reviews, however, merely involve AI- and IoT-based libraries, rather than jointly studying them (i.e., integration of AI-aided IoT as required in a “Smart Library”), see Table 1. In addition, the power of IoT technology applied in a “Smart Library”, e.g., RFID and NFC, can be remarkably enhanced when combined with AI, and AI-aided IoT could be deemed as a promising trend according to our comprehensive survey on the “Smart Library” [28,29,30,31,32,33].

In addition, we have selected the well-known and generally adopted database: SCOPUS [34], as the primary publication source to guarantee the quality and sufficient coverage of this survey. The general form of the adopted searching string is shown as follows.

*ALL ((“smart library" OR “smart libraries" OR “intelligent library" OR “intelligent libraries”) AND (“IoT" OR “Internet-of-Things”) AND (“AI" OR “artificial intelligence”)).*

**Figure 2:**
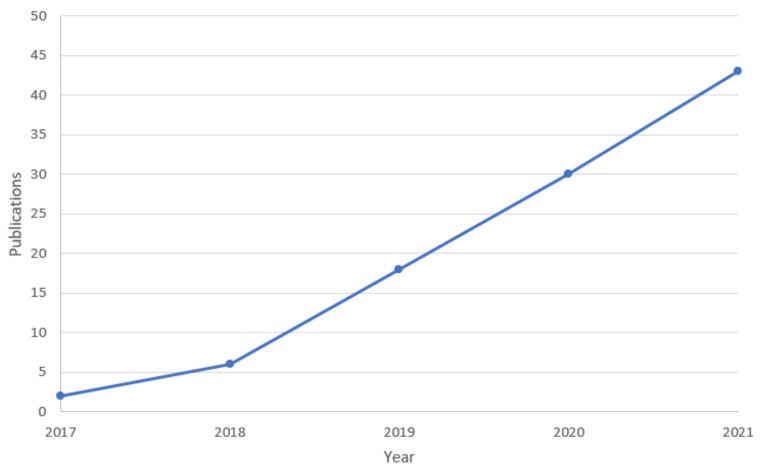
The resultant publications in the most recent five years retrieved from SCOPUS with the adopted search string [34].

As shown in Figure 2, the resultant publications retrieved from SCOPUS with the adopted search string exactly have a tendency of monotonic ascent in the most recent five years. This is in accord with the rapid development and promising prospects of AI, IoT, and corresponding applications in the smart library. Furthermore, it is worth noting that there exist amounts of cases that have not been captured under explicit keywords involved in the searching, e.g., a novel approach jointly adopting deep learning and RFID is applied in the digital library, or academic library, or public library, but without the specific words such as “smart” and “intelligent” as explicitly involved. We have thus enhanced this amount of works by thoughtfully identifying and collecting from more sources, e.g., “IEEEXplore” [35], and elaborated them in the following section as a critical supplement. From the viewpoint of readers, the fundamental functionality of the library can be subdivided into resources, space, circulation, consulting, training, acquisition, etc. [21,22,36,37]. The readers can enjoy different kinds of services provided by the library, meanwhile, from the aspect of librarianship [24,38], the traditional library itself has the responsibility for maintaining the normal operations for sustainability [24,39,40,41] and personal security [24,42,43,44,45], on both of which there is still a massive amount of room for improvements. Furthermore, the library, as a public service organization, not only needs to provide efficient services, but also needs to take the responsibility for bearing societal obligations [38,46,47] such as sustainability and personal security. The introduction of AI and IoT has indeed enhanced the efficiency of traditional service from the aspect of readers, meanwhile, it has also indeed enhanced the efficiency of responsibility for maintaining normal operations on sustainability and personal security from the aspect of librarianship itself. In addition, it is worth noting that the taxonomy of three categories (i.e., smart service, smart sustainability, and smart security) is also based on the practical retrieval results, and this can also be consistent with the directions of future development: high service efficiency, sustainability, and high demand for privacy protection and personal safety. The librarianship has been enhanced with AI-aided IoT, based on the aforementioned practical publications analysis on the existing literature.

These are the exact motivations for this review to give such a comprehensive survey for the novel, promising, and powerful domain of AI-aided IoT applied in the “Smart Library”.

### 1.3. Contributions

In this paper, we make a comprehensive overview for the AI-aided IoT applied in the “Smart Library”. The contributions of this paper can be summarized as follows:

1. We judiciously note that the promotion of IoT technology can be remarkably enhanced when combined with AI from all aspects of librarianship, and review the novel works which utilize AI-enhanced IoT to realize smarter management in the library.
2. We have given a formal definition for the “Smart Library” based on the comprehensive investigations of recent publications with both AI and IoT deployed.
3. Based on a comprehensive survey of the existing literature and practical deployments, we notify and give an overview of the constructions of the “Smart Library” in three dimensions, namely, smart service, smart sustainability, and smart security, as the main focuses of current research status.
4. We comprehensively identify the promising trend towards the future smart library, based on the recently published literature on AI-aided IoT.

The remainder of this paper is organized as follows: in Section 2, the subject is described in structure and corresponding details are presented. In Section 3, related works on either AI or IoT key techniques are classified into three aspects and specifically elaborated. In Section 4, related works on AI-aided IoT technologies applied in librarianship are comprehensively studied. The challenges and future trends are discussed in Section 5, followed by the conclusion in Section 6.

## 2. Smart Library

### Structure

**Figure 3:**
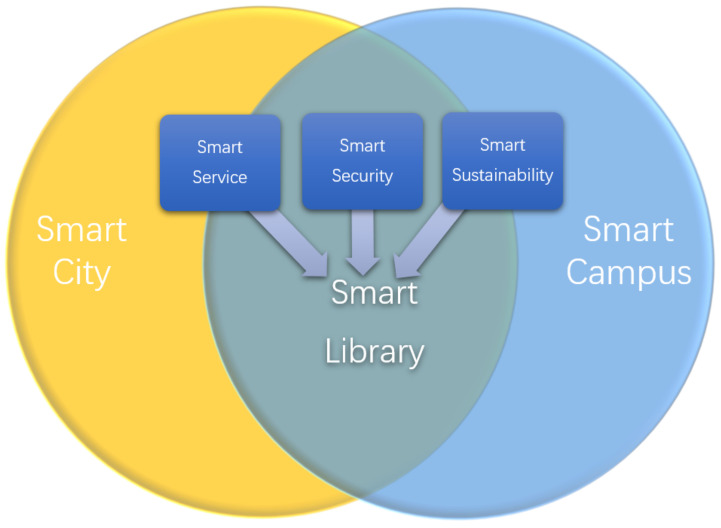
The relationship between smart city, smart library, and smart campus from the viewpoint of applying AI and IoT.

From the viewpoint of functionality, the “Smart Library” can be divided into the “smart public library” and the “smart academic library” (in most cases referring to the “smart university library”). The “smart public library”, as a key application of the “smart city”, holds the majority of the aspects of the “smart city” [23,48,49], which mainly includes “smart public service”, “smart public security” and “smart public sustainability”. In general, these features rely on AI-aided IoT as their foundation. The “smart academic library”, as a critical application of the “smart campus”, holds not only the three features mentioned above as the “smart public library”, but also the peculiar focus of the promotion of cultural education and scientific research as aforementioned. In general, we unify both the “smart public library” and the “smart academic library” as the “Smart Library”, and further review the related works from three aspects: “smart service”, “smart security”, and “smart sustainability”. We appropriately reconcile the peculiar promotion service to cultural education and scientific research to smart service. In Figure 3, we describe the relationship among the “Smart City”, the “Smart Library”, and the smart campus, from the aspect of AI and IoT applications. We highlight “smart service”, “smart security”, and “smart sustainability” as the core functionalities for both the public library and academic library.

Unfortunately, there hardly exists a formal and generally accepted definition for “Smart Library” yet. By reviewing a considerable amount of works, we define the “Smart Library” as a smart entity with AI-aided IoT technology deeply deployed to efficiently promote all aspects of operational efficiencies, for improvements of readers’ needs and sustainable social responsibilities [21,22,23,24,25,38,39,40,41,42,43,44,46,47].

The concept of a “Smart Library” was originally reported in [50] as a practical location-aware scenario, where the readers want to find the optimal route to approach the intended books in the library. The readers only need a personal digital assistant (PDA) to confirm the position of the target book(s) by the smart circulation service supported by the library. Compared to the traditional way of searching for books with manual queuing, it is much more efficient to adopt AI-aided IoT technologies [50].

## 3. Key Technologies of Smart Libraries

Since the innovative trial in [50] successfully drew attention from librarians, more IoT- and AI-based techniques have been applied in almost all aspects of librarianship, as elaborated below.

### 3.1. Fundamental IoT Technologies

IoT technologies have a deep impact on the routine management and operation of the library. For simplicity, we mainly elaborate on the core and most representative techniques.

#### 3.1.1. RFID

As a widely adopted IoT technology, RFID can trace its history back more than 70 years. Owing to the rapid growth of the integrated circuit industry, the feature size of semiconductors decreases year by year, and so does its cost. That greatly encourages RFID applications. A typical RFID system applied in a library can be divided into passive RFID tags, RFID readers, and central process devices. The identification of RFID tags can be concisely interpreted. The passive RFID tag relies on the electromagnetic induction field induced by the radio frequency signal emitted from an RFID reader to generate power for further two-way communication and data transmission. In the scenario of the smart library, the RFID technology has been widely applied in access control, book self borrowing and returning, smart shelves, etc. [51,52,53,54].

#### 3.1.2. Wi-Fi

As one of the standard configurations for almost all public infrastructure, the IEEE 802.11 standard, namely Wi-Fi, has been deeply deployed and applied in all kinds of practical indoor scenarios in modern society, e.g., in libraries, supermarkets, banks, restaurants, hospitals, etc. By resorting to Wi-Fi with powerful networking capabilities, people can conveniently connect to the internet with any smart device to realize all kinds of web-based social or business intentions. Meanwhile, Wi-Fi is reported to have a wide coverage with up to 1 kilometer (km). Although Wi-Fi is generally deployed for communication, Wi-Fi-based localization technology has already become a hot topic. The main reason for the generality of Wi-Fi-based localization is due to the localization system that can be directly built with Wi-Fi access points originally deployed for communications, without needing any extra resources [55,56,57,58,59]. In the scenario of the smart library, Wi-Fi has been widely applied in navigation for finding the books.

#### 3.1.3. BLE

As the novel version of Bluetooth, BLE has already been widely deployed and used for localization, context proximity detection, activity sensing, etc. As can be intuitively sensed from the name, the low power can be a key advantage for BLE and can thus be a natural choice for being deployed into IoT application scenarios with power constrained. In addition, BLE can have a coverage of up to 100 m, while providing a data transmission rate of up to 24 Mbps. Due to the powerful features on low power, long range coverage, affordable data rate, and low production cost, the BLE technology has an overwhelming advantage over the other solutions for some specific scenarios, e.g., localization in the power and cost constrained scenario [60,61,62,63,64]. In the scenario of the smart library, BLE can be mainly applied in navigation for finding specific places, and social interconnection for discussion and learning among the students [65].

### 3.2. Fundamental AI Technologies

There have also been a number of AI-based techniques applied in the smart library, among which the most adopted ones are concisely introduced as follows.

#### 3.2.1. NLP

NLP is a promising technology which helps the machine understand, process, and even generate human language. A machine can understand and interact with humans under advanced concepts, algorithms, and formulations defined by NLP. Thus, NLP has been widely applied in search engines, automatic question answering systems, and intelligent robots. In the scenario of the smart library, the NLP technology can be generally used in all kinds of traditionally manual techniques, e.g., a chat robot with embedded NLP technology can be deployed for consulting in the reception of library, and also for navigation systems used for finding the intended books [66,67,68,69,70,71].

#### 3.2.2. Deep Learning

Deep learning can be one of the most important machine learning (ML) technologies, which greatly impacts AI development. As a representative method, the deep neural network is powerful, with deep layers connected to form a hierarchical abstract representation structure. Deep learning-based variants include, but are not limited to, a convolutional neural network (CNN), recurrent neural network (RNN), graph neural network (GNN), as generally applied in the area of computer vision (CV), NLP, and graph-related data structure processing, respectively [72,73,74]. In the scenario of the smart library, deep learning technology can be applied in all kinds of domains with statistical data generated to learn and represent the implied features and rules [12,75,76].

#### 3.2.3. Recommender Systems

The technology of recommender systems is a practical and efficient solution to the information overload related problem. The ideology of recommender systems technology is based on a fundamental assumption that people generally rely on suggestions from others when faced with important decisions. Based on the premise, web-based retail businesses can generally adopt recommender systems for recommending people with more useful and related commodities while gaining profits via either content-based recommendations or neighborhood-based collaborative filtering (CF) recommendations. In the scenario of the smart library, the recommender systems can be used as the core engine for recommending more valuable books and research papers to the readers to improve the efficiency of operations and also the loyalties of readers [13,77,78,79,80].

## 4. AI-Aided IoT Technologies

Apparently, both IoT and AI have greatly advanced librarianship. However, relying solely on any unilateral technique still cannot realize the full potential in practice.

In a practical case of service aspect, the introduction to the RFID-based self-borrowing-and-returning machine has no doubt improved the efficiency compared with the traditional manual works of librarian staff. However, if the self-borrowing-and-returning machine is deployed in an unsuitable location, the promoted efficiency can be considerably limited without sufficient usage. Aided by AI, the management of placements of self-borrowing-and-returning machines can be appropriately scheduled and dynamically optimized by analyzing the data of historical usage and reader trajectory with AI algorithms.

In a practical case of the sustainability aspect, the high level of light brightness in the corner of the reading room can be seen as a waste of resources if the books are rarely used. With the aid of AI, the level of brightness can be smartly adjusted according to the smart analysis on historical sensing data of reader trajectories. Furthermore, for keeping a steady environment, many air conditioners that consume a considerable amount of energy are densely deployed in the reading rooms, compact stacks, and data centers. This can be a severe resource waste if the usages are insufficient. Such a low efficient sustainability consumption problem can also be suitably handled by deploying an appropriate number of air conditioners or dynamically scheduling the operation mode based on the smart analysis of the historical environment data.

In a practical case of the security aspect, the malicious borrowing transactions can be detected in advance under the smart analysis on the historical borrowing data collected from the RFID self-borrowing-and-returning machine, and subsequently trigger the further requirement of verification, e.g., check out and confirm the reminder message sent by the AI system. In general, an anomaly can be quickly detected by AI once there exists a significant deviation from the normal operation mode.

On the other hand, relying solely on AI cannot be flawless. As discussed in the earlier case, malicious borrowing transactions may be risky. The malicious person can be alarmed by the RFID access control system and subsequently trapped by security guards even if they pretend that they are a legitimate user with stolen books deeply packed in their bag when they attempt to leave out of the library directly. In another case, the historical data of readers used to feed AI algorithms cannot be collected and analysed without the help of all kinds of IoT devices [81,82,83,84].

In general, Both AI and IoT are closely interconnected and can enhance each other when applied in the constructions of the “Smart Library”. We comprehensively investigate AI-aided IoT applied in the “Smart Library” from three aspects, as follows.

### 4.1. Applications in Smart Library Service

The ultimate aim of librarianship is always to supply readers with a comfortable environment, and maximize the usage efficiency.

**Figure 4:**
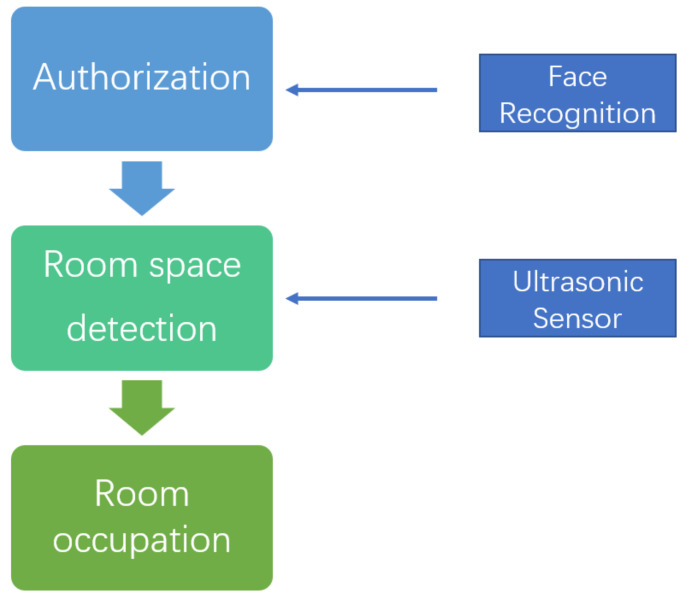
The block diagram for the process of study room occupancy [89].

The issue of seat arrangement has yet to be satisfactorily handled in traditional ways. An occupied seat without anyone there is a severe waste of resources, especially in the season of exam preparation. Some advanced approaches based on AI-aided IoT have been proposed to alleviate such dilemmas. In [85,86], the seat utilisation information data can be dynamically monitored, analysed, and managed based on a web application, pressure and RFID sensors. In [87,88], the readers can conveniently login, reserve, scan, check in, and cancel seats in the reading room solely based on a mobile device. The real-time data can be used to smartly schedule according to the dynamic needs from space usage status. The AI-aided IoT has also been applied in a similar case for study room occupancy, e.g., in [89], the room occupancy service requires a sequence of steps to fulfil the reservation process: 1. fetch access authorization based face recognition algorithm; 2. detect the occupied status via an ultrasonic sensor; 3. the authorized user can occupy the study once the room is free, based on the notification from the second step. The block diagram for the process of study room occupancy is shown in Figure 4. In [19], a very interesting positioning system solution based on the fusion of BLE beacons, Wi-Fi, and K-nearest neighbor (KNN) is proposed to facilitate the cooperation for students, optimize the space usage, and improve the efficiency of smart space service. Based on the solution provided by [19], the students can spontaneously and collaboratively form all kinds of subject study groups to facilitate orderly and efficient configuration on the usages of smart library spaces. The solution can be mainly divided into two stages: the offline stage and the online stage. In the offline stage, a Wi-Fi-based fingerprint database is created based on prior measurements of each location in the specific space in the library, e.g., a desk, a table, or a study room. In the online stage, the student can utilize a suitable smart device and the Android program provided by the suite of positioning system solution to retrieve the signals from Wi-Fi access points and send such information for querying the estimated position. The optimal estimated position can be chosen between BLE-based estimation and Wi-Fi-based estimation based on the comparisons of error probability density. Here, it is worth noting that the estimation by the Wi-Fi-based approach is estimated by the most common K nearest locations stored in the fingerprint database, based on the KNN algorithm. The one with the smallest error probability density between the BLE-based estimation and the Wi-Fi-based estimation is chosen as the final predicted position for a specific student. Meanwhile, the online real-time predicted position can also be used to update the offline fingerprint database. Once the accurate position is determined via the positioning system solution, the student can readily advertise his/her subject interest and position to all the others via labeling or highlighting on the visualization map also supported by the solution, and those with the same or similar subject interest will easily take note and further form a set of interactive subject discussion groups. The usage of space can thus be optimized, and the efficiency of smart space service can also be remarkably improved.

**Figure 5:**
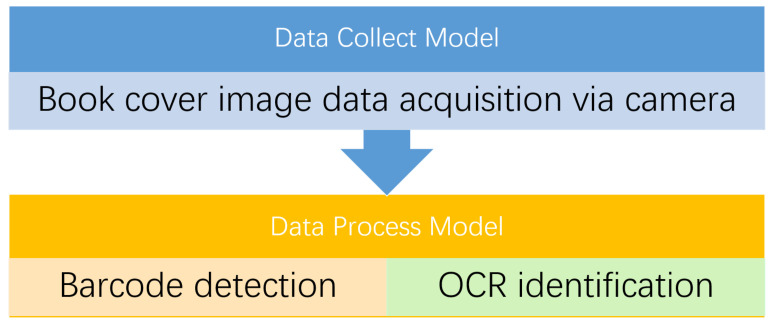
The architecture of smart book sorting system [90].

The book sorting system is a fundamental and important tool for improving the efficiency of the book circulation service. In [90], the efficiency is remarkably improved with barcode and deep learning-based optical character recognition (OCR) deployed. The architecture of the smart book sorting system by [90] is composed of two main parts: Data Collect Model and Data Process Model, as shown in Figure 5. The Data Collect Model is mainly used for the book cover image data acquisition via camera. The Data Process Model can subsequently process the detection and identification of barcodes with deep learning-based OCR. In [91], a drone robot is utilized for book inventories. Based on the visual localization and OCR, the tags on the books can be identified by the drone robot and further used for inventory service. In [13], a novel approach based on cloud and recommender systems is proposed to recommend students more interesting and useful books. This approach not only allows the readers to rate the borrowed books but also receives recommended books based on the historical data stored in the cloud. The whole suite of systems by [13] can be time-saving and cost-saving for readers. In [92], a novel library audio approach using OCR, deep learning, and ultrasonic sensors is proposed to help blind people hear and comprehend the content of books. In detail, firstly, the ultrasonic sensor is utilized to help determine the distance between the OCR device and book, for the sake of better recognition in the next step. Secondly, the OCR combined with deep learning is utilized to recognize the content of the physical book and then convert it to text content. At last, the text content is further converted to the corresponding audio file and then played via the headset. This can efficiently help blind people read physical books. In [93], a novel model for improving the network teaching is proposed. This model utilizes the interactive and feedback data generated in the class of network teaching to analyse statistical rule based machine learning, further updating the corresponding data stored in the cloud. The proposed approach can provide a scientific evaluation on the individual cognitive feature for students.

In [94], a suite of solutions is proposed for the problem of misreading RFID tags in the circulation service. Based on the prior measurements of received signal strength (RSS) combined with KNN, an accurate localization of RFID tags of books can be used for distinguishing the needed books from misread ones. Similarly, the approach proposed by [95] utilizes the RFID and machine learning algorithm to localize the books preserved in the cabinet. This approach can efficiently improve the accuracy of the determination of the books preserved in the row, cabinet, and rack. The approach proposed by [96] is also utilized to localize the books preserved in the bookshelf, based on RSS collected from RFID and deep learning.

**Figure 6:**
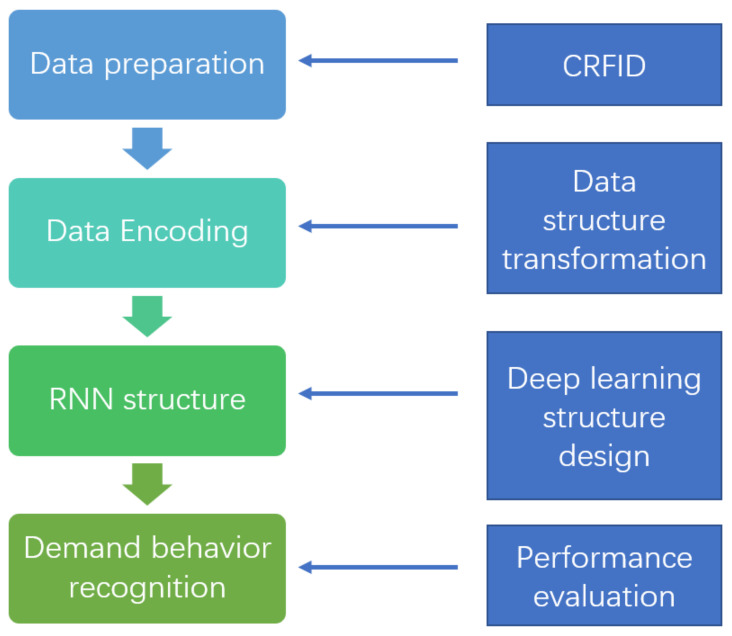
The workflow of the personalized activities learning system [97].

In order to improve the personalized service of user needs for books, the readers’ degree of need for the book can be an important metric for evaluating the efficiency of the book usage service. In [97], RNN deep learning aided computational RFIDs (CRFID) technology is utilized to detect and identify the reading activities of readers, such as picking up the book, browsing the title, skimming through the page, moving the location of the book, reading, and borrowing. The activities data are collected via CRFID, and the collected data naturally possess sequential features and are specially fitted for the RNN-based technology for machine learning, and further provide constructive suggestions on satisfying the needs of the readers. The workflow of [97] is shown in Figure 6.

In summary, [20,85,86,87,88,89,90,91,94,97] fall into categories of AI-aided IoT applied in smart space service, smart learning service, smart circulation service, and smart acquisition service, respectively. For the sake of conciseness, the representative smart service-related literature is summarized in Table 2.

### 4.2. Applications in Smart Library Sustainability

**Figure 7:**
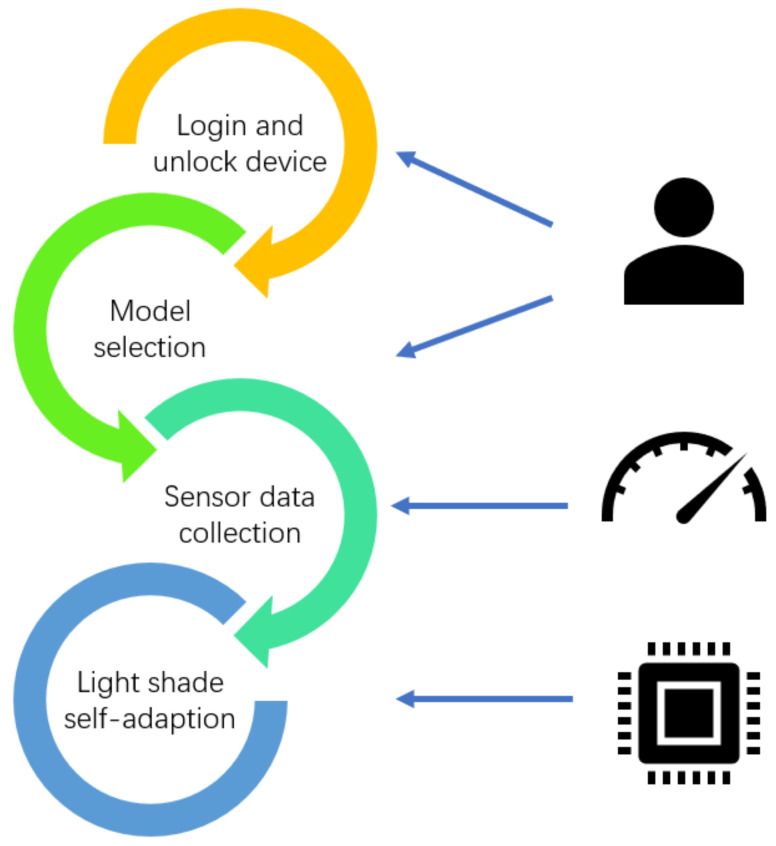
The the workflow of the smart light shade control system [99].

**Figure 8:**
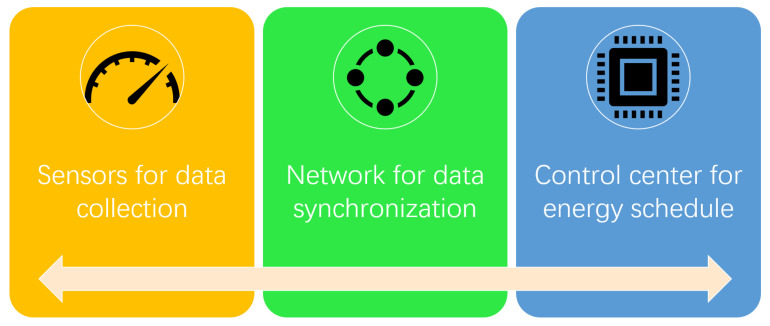
The workflow of the smart sustainability control structure [100].

The carbon footprint has always been a significant issue which relates the sustainable development of human society [98]. Any library needs to consume a considerable amount of resources for routine operations and management. In addition, the library also discharges a significant amount in the product of metabolism including carbon footprint. It is thus in urgent need of sustainable management of resource consumption. By resorting to AI-aided IoT, sustainability can be smartly scheduled according to practical needs. It is reported in [99] that a suite of AI-aided IoT solutions is proposed to handle light shade in the library to maximize the usage efficiency of natural light. The reader can log in to the device control system and choose the smart mode. The sensors start the detection of the height and angle of light and become self-adaptive to the parameters set by the readers, as shown in Figure 7. In [100], a framework of sustainability consumption management system for the smart library is proposed to maximize the sustainability usage efficiency. The framework consists of multi-source sensors, a sensor network, and a server, where sensors are used to collect data such as temperature, humidity, personal information, etc., as shown in Figure 8. The collected data are synchronized with each other and with the server, and based on the historical data from sensors. The control center deployed in the server can undertake smart planning for equipment usage, e.g., the light system can be dynamically scheduled according to the time series data analysis. In [101], a novel preservation solution aiming at preserving the environmental conditions is proposed. This solution can provide better preservation for the environmental conditions, with the help of a multi-sensor and monitoring data visualization. In summary, we can unify [99,100,102,103,104,105,106,107] as the applications of AI-aided IoT applied in smart basic service. For the sake of conciseness, the distinctive smart sustainability-related literature is summarized in Table 3.

### 4.3. Applications in Smart Library Security

**Figure 9:**
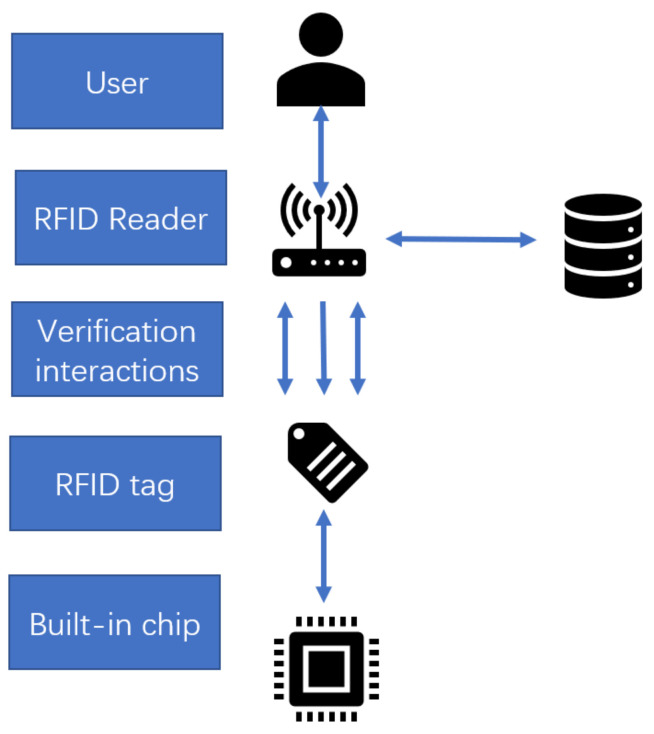
The workflow of smart RFID authentication system structure [108].

**Figure 10:**
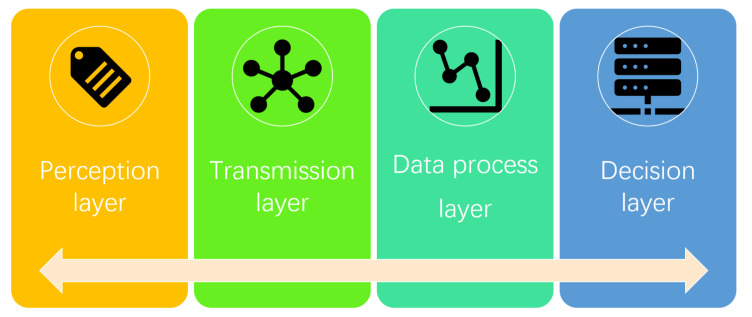
The workflow of AI-enhanced IoT warning system structure [109].

People can enjoy the convenience and comfortable reading experience provided by library. Meanwhile, personal belongings and private information are exposed to risk consciously or unconsciously in such a public scenario. Furthermore, with the coming of the significant data era and corresponding processing algorithms, the needs and desires for privacy protection will intensify. By resorting to AI-aided IoT, the risk of exposure of personal belongings and personal privacy can be effectively mitigated. It is reported in [108] that an innovative authentication structure is proposed to handle the protection of the readers’ privacy. In [108], the data collected by RFID is encrypted with an intelligent authentication algorithm, and the secure transaction can be fulfilled with several data interactions by the proposed authentication algorithm, as shown in Figure 9. In [109], an IoT risk warning system structure with case-based reasoning and enhanced fuzzy sets is proposed to monitor the status of all kinds of devices automatically, along with the reader activities. The workflow of the proposed AI-enhanced IoT warning system structure in [109] consists of several steps: 1. The sensors detect and identify the risks of anomalies. 2. The data collected by the sensors are then transmitted to the data processing and evaluation layer according to the IoT-based techniques and protocols. 3. The output data are then compared with the stored historical anomaly event data in the processing and evaluation layer. 4. The evaluated information is then sent to the decision layer based on case-based reasoning and fuzzy sets for the final decision on the detected anomaly event. 5. After the decision by the decision layer is made, the stored historical data are further updated and stored for the subsequent usages on data process and comparison, as shown in Figure 10. In summary, works [108,110,111] can be unified as the applications of AI-aided IoT applied in innovative circulation services. Works [112,113,114] can be unified as the applications of AI-aided IoT used in smart digital resource service. Works [109,115,116] can be unified as the applications of AI-aided IoT applied in the smart basic service. For the sake of conciseness, the specific smart security-related literature is summarized in Table 4.

## 5. Challenges and Open Directions

Despite the considerable progress in AI-aided IoT applied in smart libraries, there is still room for further improvement.

From the aspect of smart service, the evaluated performance, such as accuracy and recall of the existing solutions for innovative recommendation-based scenarios, is comparatively low. More advanced NLP technologies such as Bert [69] and graph-based neural networks [2,117,118,119] with side information enhanced could be deployed as the important upstream or downstream engine for data processing when combined with IoT in the near future.

From the aspect of sustainability, the deployed cost of sensors for sensing environment data is still high. It is thus unpractical to pervasively implement all kinds of monitor devices in the existing solutions for the smart sustainability of the library. This defect could be efficiently solved under the cooperative and coordinated framework of a wireless sensor network (WSN) [120,121]. Thus, there is ample scope for embedding the AI-based algorithms into such collaborative WSNs. Meanwhile, the expense and size of a single sensor product could rationally diminish according to the well known Moore’s Law. This signifies that, more and more, sensors can be practically affordable and deployed to intensify service performance in the near future.

From the aspect of smart security, the existing solutions are inclined to deal with the homogeneous data generated by specific sensors, e.g., RFID. However, with the diversification of business scenarios for the smart library, the joint coordinated processing of heterogeneous data generated from multi-purpose sensors such as RFID, NFC, bluetooth, infrared, etc., could be a promising trend. How to optimally cope with the amount of sensed multi-source data with interrelated redundancy is a big challenge [122]. In addition, generated by massive IoT devices, the large-scaled data magnifies the importance of privacy protection. In particular, the heterogeneous and multi-source collected data in smart library scenarios involve a considerable amount of personal and privacy data (e.g., facial image data), and the leakage of such critical data will have a strong impact on people’s lives. As such, it is remarkably imperative to pay attention to such key issues on privacy data security, and this mainly involves two critical and fundamental properties of information security: data confidentiality and data integrity. From the aspect of data confidentiality, the symmetrical key encryption algorithm can be efficiently utilized to protect the key information from being stolen in the process of both data transmission and data storage. In addition, the access control can also be an efficient way for preventing unauthorized disclosure of private information. As for the data integrity, the related security mechanisms mainly involve the secure hash algorithm. For example, the hash data integrity checking algorithm can be efficiently used to protect the data from the malicious authorized modification, by converting the privacy information into a fixed length of data for the further verification of hash values in the process of both data transmission and data storage [123,124,125,126]. Under the discussed secure mechanism scheme, the collected heterogeneous and multi-source data can be preserved and further dependably used for downstream secure data analysis, e.g., the distributed multi-party training approach.

The federated learning-based AI algorithms [127,128,129] can be profoundly utilized to facilitate the data exposure risk concealed in the existing centralized training solutions adopted by librarianship. Last but not least, with the introduction of a series of concepts, e.g., Digital Twins [130,131] and Metaverse [132], the smart library with such refreshing technologies could be promising in the near future.

## 6. Conclusions

In this review, we have conducted a comprehensive survey on AI-aided IoT technologies in emerging smart libraries, aiming to explicitly provide a systematic, structured and profound scheme for such a promising field. A practical and concrete case of smart circulation service demonstrates that a considerable amount of improvements on the service have been brought by the paradigm of the smart library with the help of AI and IoT, in contrast to the traditional human-based librarianship. Although there are extensive application scenarios with AI and IoT deployed in a smart library, we focus on core aspects: smart service, smart sustainability, and smart security. We have discussed the derivation of the smart library and have given a formal definition of the smart library based on extensive surveys of the state-of-the-art literature. We have provided a brief introduction on the key technologies of AI and IoT applied in the smart library, which can be the critical foundation for the following sections. We have conducted a comprehensive review of the state-of-the-art applications in the smart library with AI and IoT solutions deeply deployed. We have summarized the challenges and promising future directions, for the sake of inspiring readers with emerging topics applied in the field of the smart library.

## Figures and Tables

**Table 1:** Comparison between our and existing surveys.

Compared Survey	IoT Involved	AI Involved	AI-Aided IoT Integration
Ozeer (2019) [21]	✓		
Cao (2018) [22]	✓	✓	
Gul (2019) [23]	✓	✓	
Schöpfel (2018) [24]		✓	
Asemi (2021) [25]		✓	
This survey	✓	✓	✓

**Table 2:** The surveyed literature on AI-aided IoT applied in smart library service.

Scenario in Library	Related AI-Aided IoT	Year [Ref.]
Space service	Sensors + smart arrangement	2019 [85], 2021 [86]
Space service	Mobile device + smart arrangement	2019 [88], 2021 [87]
Space service	Sensors + face recognition	2019, [89]
Space service	Sensors + KNN	2016, [19]
Circulation service	Sensors + computer vision based OCR	2017 [20]
Circulation service	Sensors + deep learning based OCR	2021 [90,91]
Learning service	Sensors + computer vision based OCR	2021 [92]
Learning service	Cloud + machine learning	2021 [93]
Learning service	cloud + recommender system	2020 [13]
Circulation service	Sensors + KNN	2021 [94]
Circulation service	sensors + KNN/SVM	2020 [95]
Circulation service	sensors + deep learning	2020 [96]
Acquisition service	Sensors + RNN	2020 [97]

**Table 3:** Existing studies on AI-aided IoT applied in smart library sustainability.

Related AI-Aided IoT	Year [Ref.]
Sensors + self-adaption	2021 [99]
Sensors + smart schedule	2019 [100]
Mobile terminal + smart schedule	2017 [102]
Sensors + smart monitoring	2021 [103]
Sensors + smart harvesting	2017 [104]
Sensors + smart monitoring	2021 [105,106]
Multi-sensor + smart preserving	2019 [101]
Sensors + image classification algorithm	2021 [107]

**Table 4:** The surveyed literature on AI-aided IoT applied in smart library security.

Application Scenario in Library	Related AI-Aided IoT	Year [Ref.]
Smart circulation service	Sensors + smart authentication	2020 [108]
Smart basic service	Sensors + case-based reasoning	2019 [109]
	and fuzzy sets	
Smart basic service	Sensors + machine vision	2020 [115]
Smart circulation service	Sensors + smart identification	2016 [110]
Smart digital resource service	Cloud + smart identification	2022 [112]
Smart basic service	Trust network + smart encryption	2019 [116]
Smart circulation service	Sensors + smart attacks prevention	2021 [111]
Smart digital resource service	Sensor network + intelligent	2020 [113]
	contract prevention	
Smart digital resource service	Cloud + smart encryption	2021 [114]
